# CCR10/CCL27 crosstalk contributes to failure of proteasome-inhibitors in multiple myeloma

**DOI:** 10.18632/oncotarget.12522

**Published:** 2016-10-08

**Authors:** Shanmugapriya Thangavadivel, Claudia Zelle-Rieser, Angelika Olivier, Benno Postert, Gerold Untergasser, Johann Kern, Andrea Brunner, Eberhard Gunsilius, Rainer Biedermann, Roman Hajek, Ludek Pour, Wolfgang Willenbacher, Richard Greil, Karin Jöhrer

**Affiliations:** ^1^ Tyrolean Cancer Research Institute, Innsbruck, Austria; ^2^ Laboratory of Tumor Angiogenesis and Tumorbiology, Department of Internal Medicine V, Medical University of Innsbruck, Innsbruck, Austria; ^3^ Department of Pathology, Medical University Innsbruck, Innsbruck, Austria; ^4^ Department of Orthopedic Surgery, Medical University Innsbruck, Innsbruck, Austria; ^5^ Babak Myeloma Group, Department of Pathological Physiology, Faculty of Medicine, Masaryk University, Department of Clinical Hematology, University Hospital Brno, Brno, Czech Republic, Department of Hematooncology, Faculty of Medicine, University of Ostrava and University Hospital Ostrava, Ostrava, Czech Republic; ^6^ Department of Internal Medicine V, University Hospital Innsbruck, Innsbruck, Austria; ^7^ Salzburg Cancer Research Institute-Laboratory of Immunological and Molecular Cancer Research, Salzburg, Austria; ^8^ Third Medical Department at The Paracelsus Medical University Salzburg, Austria; ^9^ Cancer Cluster Salzburg (CCS), Salzburg, Austria

**Keywords:** myeloma, CCR10, CCL27, drug resistance, stroma cells

## Abstract

The bone marrow microenvironment plays a decisive role in multiple myeloma progression and drug resistance. Chemokines are soluble mediators of cell migration, proliferation and survival and essentially modulate tumor progression and drug resistance. Here we investigated bone marrow-derived chemokines of naive and therapy-refractory myeloma patients and discovered that high levels of the chemokine CCL27, known so far for its role in skin inflammatory processes, correlated with worse overall survival of the patients. In addition, chemokine levels were significantly higher in samples from patients who became refractory to bortezomib at first line treatment compared to resistance at later treatment lines.

*In vitro* as well as in an *in vivo* model we could show that CCL27 triggers bortezomib-resistance of myeloma cells. This effect was strictly dependent on the expression of the respective receptor, CCR10, on stroma cells and involved the modulation of IL-10 expression, activation of myeloma survival pathways, and modulation of proteasomal activity. Drug resistance could be totally reversed by blocking CCR10 by siRNA as well as blocking IL-10 and its receptor.

From our data we suggest that blocking the CCR10/CCL27/IL-10 myeloma-stroma crosstalk is a novel therapeutic target that could be especially relevant in early refractory myeloma patients.

## INTRODUCTION

Multiple myeloma is an incurable plasma cell malignancy characterized by the clonal proliferation of plasma cells in the bone marrow compartment. This specific microenvironment constitutes a sanctuary for the cancer cells supporting growth, survival and drug-resistance via cell-to-cell contact and soluble mediators (reviewed in [1]). The application of novel therapeutic agents such as proteasome inhibitors and immunomodulatory drugs, which not only target the malignant clone but also the supportive microenvironment led to improved progression-free and overall survival. Yet, myeloma patients still acquire resistance to all available therapies and finally succumb to their lethal disease.

Chemokines are a family of low molecular weight (8–10 kDa) secreted, homeostatic or proinflammatory cytokines that regulate cell trafficking via interacting with a subset of seven-transmembrane G-protein coupled receptors [2]. These chemotactic cytokines are also major players in the crosstalk between tumor cells and stroma cells and are essentially involved in metastasis, survival, development of drug resistance and escape from immune surveillance in numerous cancer entities [3–6]. Chemokines in multiple myeloma are known to contribute to the bone marrow homing of the malignant cells and ligand binding can also modulate integrin expression contributing to the development of cell adhesion-mediated drug resistance [7–9]. In addition, osteolysis, a main characteristic of advanced myeloma disease, is essentially mediated by chemokines [10]. However, a complete chemokine profile of the diseased bone marrow is still lacking and might give clues for the development of novel therapies.

CCL27, also called cutaneous T-cell attracting chemokine (C-TACK), is mostly known to cause T-cell mediated skin inflammation [11, 12] and high serum levels are of prognostic relevance in patients suffering from psoriasis and atopic dermatitis [13, 14]. The respective receptor, CCR10, is expressed on T-cells and on various subtypes of normal plasma cells. The mature B cells respond exclusively to the second ligand of the receptor, CCL28. Signaling leads to a selective accumulation of normal IgA+ plasmablasts in mucosal tissue [15–17] and of IgE+ plasmablasts in inflamed airways [18]. In addition, CCR10 ligation by CCL28 supports the maintenance of memory B-cells and contributes to survival and proliferation of long-lived IgA-producing plasma cells [19, 20]. Recently, work of Karlsson and colleagues revealed that the CCL28/CCR10 axis is an important growth and survival pathway for hematopoietic stem cells and progenitor cells in the bone marrow niche [21]. Of note, mesenchymal stem cells and bone marrow derived keratinocytes as well utilize CCR10/CCL27 signaling [22, 23].

In this study, we investigated bone marrow-derived chemokines of myeloma patients with a special focus on early and late refractory patients. We discovered that high CCL27 levels correlated with shorter overall survival of the patients and with early resistance to bortezomib treatment. We further investigated the involved mechanisms *in vitro* and *in vivo*.

## RESULTS

### High levels of CCL27 in the bone marrow correlate with poor overall survival and early refractory disease of myeloma patients

**Figure 1:**
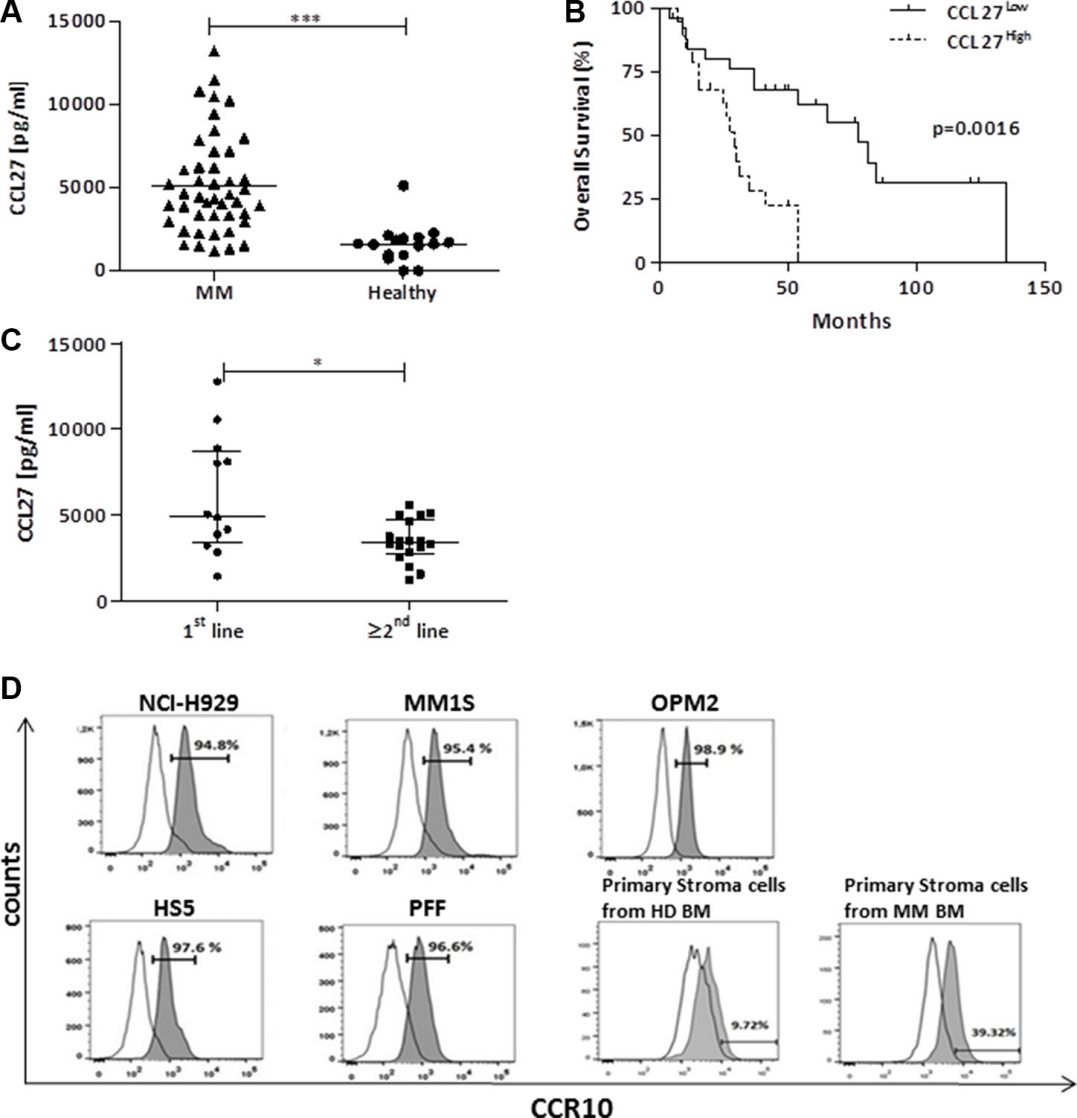
High bone marrow CCL27 levels correlate with poor survival and primary refractory disease and stromal CCR10 expression might facilitate signaling (**A**) Plasma samples from bone marrow aspirates of myeloma patients and healthy, age-matched donors (collected at Innsbruck Hospital) were analyzed for CCL27 by Elisa. Values are in pg/ml, ***p < 0.001. (**B**) Kaplan-Meier survival curves for patients expressing CCL27 at high and low levels, respectively (cutoff determined by ROC analysis). (**C**) Bone marrow plasma samples from patients refractory to bortezomib at first line treatment versus later lines were collected at diagnosis at Brno Hospital and further analyzed by Elisa as above. Boxplots show median and interquartile range. *p < 0.05; (**D**) Histograms of CCR10 expression on myeloma cell lines (NCI-H929, MM.1S, OPM-2), stroma cell line HS-5, primary fibroblasts (PFF), primary stroma cells isolated from a healthy donor (HD) and a diseased bone marrow (MM), percentage of positive cells is depicted. Open histogram: isotype control, solid histogram: specific CCR10 staining.

We screened for chemokines in bone marrow aspirates of patients and age-matched healthy donors using semi-quantitative protein arrays. Beside already known myeloma-associated chemotactic molecules, CCL27 was overexpressed in patients' samples. Concomitant Elisa analysis confirmed significant differences between myeloma (*n* = 45; median 4640 pg/ml; IQR 3320-7291) and healthy donor samples (*n* = 16; median 1620 pg/ml; IQR 947-1996; *p* < 0.0001, Figure 1A). Patients' data is summarized in Table 1. Utilizing cutoffs determined by receiver operating characteristics (ROC) analysis, we found that high levels of CCL27 were associated with worse overall survival of patients (Figure 1B; cutoff value = 4884 pg/ml; median survival 29 vs. 77 months, *p* = 0.0016). We performed multivariate analysis including CCL27 expression (high or low), sex, and stage (stage MM3B versus all other stages) as covariates. From the 45 cases, one was excluded due to missing values. Although sample numbers were low, Cox regression analysis revealed that CCL27 was an independent prognostic factor for overall survival with a hazard ratio of 4.3 [1.727 – 10.975; 95% CI, *p* = 0.002]. Of note, CCL27 levels did not correlate with tumor load (data not shown).

**Table 1:** Patients' characteristics

		Number of patients
**age**	< 65	23
	> 65	22
**sex**	Male	29
	Female	16
**ISS staging**	I	6
	II	15
	III	13
	Unknown	11
**Durie & Salmon staging**	IA	1
	IIIA	31
	IIIB	12
	Unknown	1
**Immunoglobulin subtypes**	IgG	15
	IgA	14
	IgM	1
	Light chains	11
	Unknown	4

This table displays data on patients whose naive bone marrow plasma samples were utilized for CCL27 Elisa. Data refers to the date of diagnosis.

We further analyzed untreated bone marrow plasma from patients who showed bortezomib-refractory disease at first line treatment (*n* = 12) compared to patients that became refractory to bortezomib at higher treatment lines (*n* = 18) Clinical characteristics of patients is summarized in Table 2. In a subset of first line refractory patients, CCL27 levels were significantly enhanced (Figure 1C; 1st line median 4935 pg/ml; IQR 3376-8669; other lines median 3385 pg/ml; IQR 2754-4688; *p* < 0.05).

**Table 2:** Characteristics of patients refractory to bortezomib

		Number of patients
**age**	< 65	13
	> 65	17
**sex**	Male	19
	Female	11
**refractory to bortezomib**	at 1st line	12
	2nd line and higher	18
**ISS staging**	I	6
	II	11
	III	12
	unknown	1
**Durie&Salmon staging**	IA	5
	IIA	7
	IIIA	12
	IIIB	6
**Immunoglobulin subtypes**	IgG	16
	IgA	6
	Light chains only	7
	Nonsecretory	1

Clinical characteristics of patients who became refractory to bortezomib in the course of treatment. Data refer to date of diagnosis.

CCL27 binds uniquely to its respective receptor, CCR10, which has also a second ligand, CCL28. To gain more knowledge about possible interactions of this receptor/ligand(s) system, we measured CCL28 plasma levels as well as receptor expression on plasma cells and stroma cells. Compared to CCL27, CCL28 levels were substantially lower and even not detectable in 20/42 patients' bone marrow samples (Supplementary Figure 1A; MM median 25 pg/ml; IQR 0-245; Healthy median 349 pg/ml; IQR 282-466). We confirmed previously reported CCR10 expression on myeloma cell lines and primary plasma cells [24] in our samples and we found substantial CCR10 expression on stroma cell line HS-5 and primary fibroblasts, which we routinely use as coculture systems. Primary stroma cells isolated from the bone marrow of healthy and diseased individuals also expressed CCR10. Representative flow cytometer analyses are depicted in Figure 1D. We also detected CCL27 in the supernatants from myeloma cell lines as well as stroma cells (Supplementary Figure 1B).

Functionally, Nakayama et al. showed that CCL27 and CCL28 can induce chemotaxis of myeloma cell lines [24]. We found enhanced adhesion (Supplementary Figure 2A) and chemotaxis (Supplementary Figure 2B) in response to CCL27 for myeloma cell lines NCI-H929 and MM.1S but not for OPM-2. None of the cell lines migrated in response to the CCL28 concentrations tested and both chemokines had no impact on myeloma cell proliferation (Supplementary Figure 2C). In all cell lines, the extent of bortezomib-induced cell death was not altered by the addition of CCL27 or CCL28 (Supplementary Figure 2D). Since CCL27 is primarily known for its T cell attracting properties, we additionally investigated a possible correlation between infiltrating T cells and CCL27 levels in myeloma patients on the basis of CD62L/CD45RA expression. However, T cell subset infiltration rates did not correlate with CCL27 levels (data not shown).

### In the presence of stroma cells, CCL27 rescues myeloma cells from apoptosis induced by proteasome inhibitors

**Figure 2:**
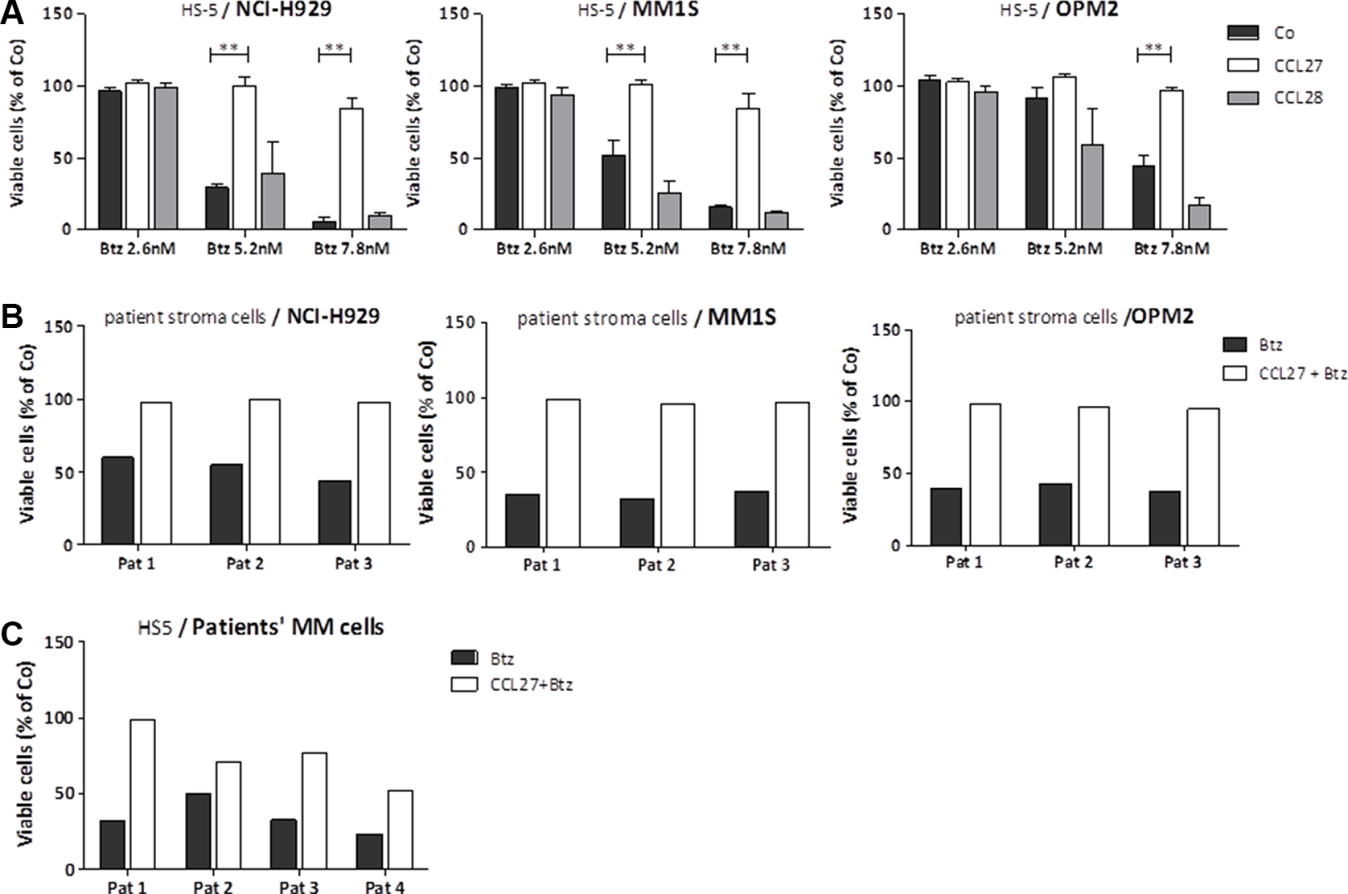
CCL27 rescues myeloma cells from treatment with proteasome inhibitors in the presence of stroma (**A**) Cocultures of myeloma cells and HS-5 stroma cells (ratio 2:1) were treated for 48 hrs with different concentrations of bortezomib (2.6/5.2/7.8 nM) with and without CCL27 (7.9 nM) and CCL28 (8.1 nM) (*n* > 3). Percentage of viable myeloma cells (Ann-V/7-AAD negativ) compared to untreated control is shown in all graphs in this figure. **p < 0.01; (**B**) Myeloma cell lines were cocultured on primary stroma cells isolated from myeloma bone marrow aspirates of 3 patients, treated as above (bortezomib 5.2 nM) and viability of myeloma cells was measured. (**C**) Similar, primary myeloma cells (CD138-sorted) from 4 different patients were cocultured with HS-5 stroma cell line, treated as above (bortezomib 7.8 nM) and their viability was measured; assays were performed in duplicates for (B) and (C).

We further utilized stroma-myeloma cell cocultures in order to approach the *in vivo* crosstalk more closely and treated the cells with different drugs. In the presence of HS-5 stroma cells, the addition of CCL27 rescued myeloma cells almost completely from bortezomib-induced cell death. Supplement of the second ligand, CCL28, had no effect (Figure 2A). Results were confirmed using primary fibroblasts (Supplementary Figure 3A). While CCL27 also blocked the induction of cell death by other proteasome inhibitors, i.e. MG-132 (Supplementary Figure 3B) and carfilzomib (Supplementary Figure 3C), efficacy of melphalan treatment was not affected (Supplementary Figure 3D). Primary stroma cells isolated from three myeloma patients also rescued myeloma cell lines (Figure 2B), and survival of CD138-sorted primary myeloma cells from four patients seeded on HS-5 layer and treated with bortezomib was ameliorated by the addition of CCL27 (Figure 2C).

### CCL27 protects myeloma cells from bortezomib treatment in a xenotransplant model

Chemokines are important players throughout all species, however, caution is requested for the interpolation of experimental results between genera, especially between mouse and human (reviewed in [25]). Instead of a mouse model, we here chose a chick chorioallantoic membrane (CAM) assay modified for the use with human myeloma cells as previously reported [26, 27]. This model has also been successfully utilized to investigate tumor-stroma interactions in other human B-cell malignancies [28, 29].

**Figure 3:**
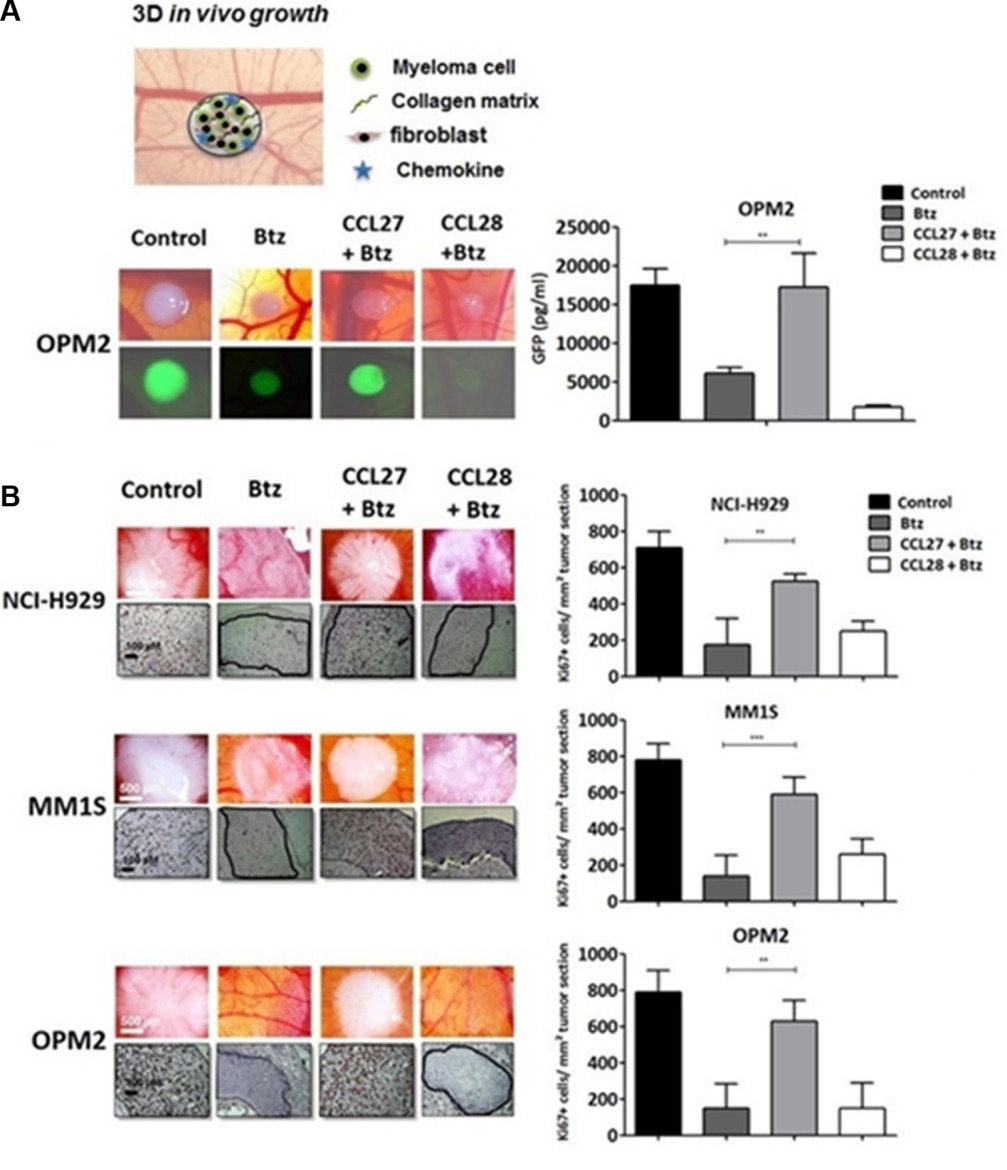
CCL27 rescues myeloma cells from bortezomib-induced cell death in a xeno-transplanted model (**A**) Chicken chorioallantoic membrane assay was used for *in vivo* testing and the setup of the 3D-onplant is summarized in the drawing. Tumor onplants containing myeloma cells and primary fibroblasts in a ratio of 11:1, bortezomib (1 nM) and/or chemokine (CCL27: 7.9 nM, CCL28: 8.1 nM) in a collagen matrix were positioned on the membrane and incubated for 5 days. Single myeloma xenografts were photographed using a stereo-fluorescence microscope (Olympus SZX10 at 12× magnification), excised, and GFP concentrations of single tumors were measured by Elisa. Values are shown as mean concentration in pg/ml ± SEM (*n* = 12). (**B**) Immunohistochemical analysis of cross-sections of NCI-H929, MM.1S and OPM-2 grafts in the chorioallantoic membrane model including control with and without bortezomib, CCL27 or CCL28 at the above mentioned concentrations; sections were stained with an anti-human Ki-67 antibody to detect growing myeloma cells. Pictures were taken using Zeiss Axiovert 200 M microscope at 200× maginification. The amount of Ki67 positive cells/tumor area are summarized in the graphs. **p < 0.01 and ***p < 0.001.

Onplants containing eGFP-transfected OPM-2 myeloma cells and primary human fibroblasts in a collagen matrix were positioned onto the CAM and treated with bortezomib with/without the presence of chemokine for five days (Figure 3A, schematic view). Tumors were excised and tumor mass was quantified using an anti-GFP-Elisa. As shown, addition of CCL27 rescued myeloma cells efficiently from treatment in this setting (Figure 3A, pictures and graph). This experiment was confirmed using NCI-H929, MM.1S and untransfected OPM-2 cells (Figure 3B, pictures and graphs). In this setting, the number of proliferating myeloma cells was assessed by immunohistochemical staining with human Ki-67. Whereas CCL27 rescued myeloma cells from drug-treatment, addition of CCL28 had no impact on cell survival in both assays.

### CCR10 ligation regulates stromal IL-10 levels and blocking the receptor restores drug sensitivity

**Figure 4:**
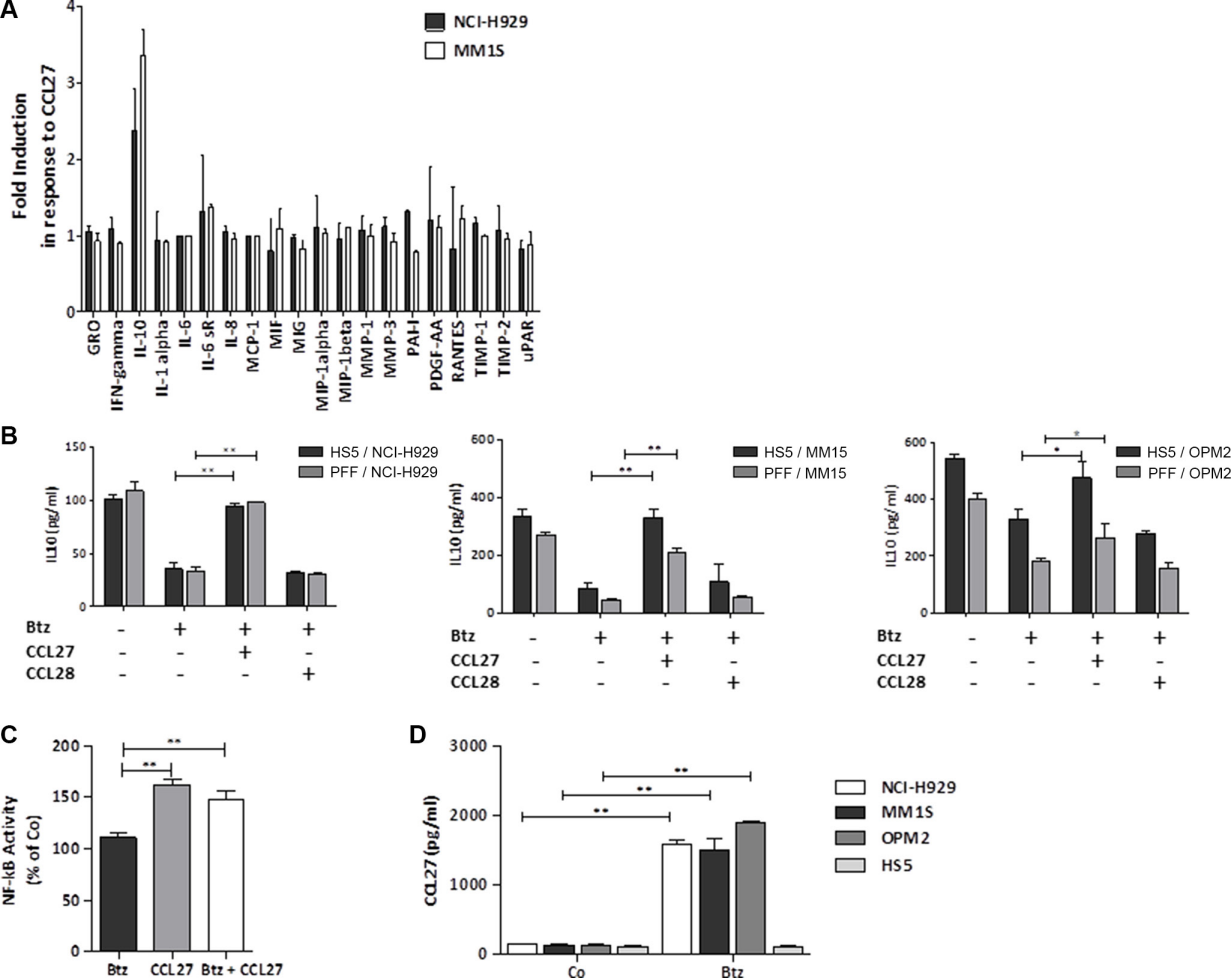
IL-10 levels remain high in treated cocultures due to CCL27 (**A**) Supernatants from cocultures of myeloma cells and stroma cells (ratio 2:1, incubation time 24 hrs) treated +/− bortezomib (5.2 nM) and CCL27 (7.9 nM) were used for protein arrays to detect soluble players involved in CCL27-induced bortezomib resistance. Fold expression under bortezomib treatment plus CCL27 compared to bortezomib alone is shown. (**B**) In the same setting, IL-10 levels in cocultures of myeloma cells and HS-5 or PFF cell layers (ratio 2:1) with or without bortezomib, CCL27, and CCL28 were assessed by Elisa. Results are shown as mean pg/ml ± SD from at least 3 independent experiments. *p < 0.05, **p < 0.01; (**C**) HS-5 stroma cells were treated for 3 hrs with bortezomib (7.8 nM), CCL27 (7.9 nM), or both, and NF-κB activity in nuclear extracts was measured by Elisa. Values are shown as percentage activity compared to untreated control. **p < 0.01 (**D**) Indicated cell lines were treated with/without bortezomib (5.2 nM) for 24 hrs and CCL27 expression was measured by Elisa (Co=control; *n* = 3; mean pg/ml +/−SD is shown).

We found that myeloma cells were rescued even when separated from the stroma cell layer by 0.4 μm transwells, although to a lower extent (data not shown). Therefore, we further focussed on soluble factor(s) that could be involved in this drug resistance. From almost 100 proteins tested (including cytokines, chemokines and growth factors), about 20 were expressed at significant levels. Out of them, one factor was significantly regulated in myeloma-stroma cell coculture treated with bortezomib +/− CCL27, i.e. IL-10 (Figure 4A).

IL-10 is discussed as a survival factor for myeloma cells [30] and is induced in myeloma-stroma coculture [31], which we also confirmed. Bortezomib treatment has been shown to inhibit IL-10 expression probably via blocking NF-κB signaling [32]. In our experiments utilizing HS-5 as well as primary fibroblasts (PFF), CCL27 prevented the bortezomib-induced downregulation of IL-10. The second ligand, CCL28, had no impact on IL-10 levels (Figure 4B). We also found that CCL27 induced NF-κB activity in stroma cells, and levels remained high when cells were treated with bortezomib and CCL27 (Figure 4C). However, CCL27 did not induce NF-κB activity in myeloma cell lines (data not shown). On the other hand, bortezomib treatment led to enhanced CCL27 expression in myeloma cell lines, but not in HS-5 stroma cells (Figure 4D).

**Figure 5:**
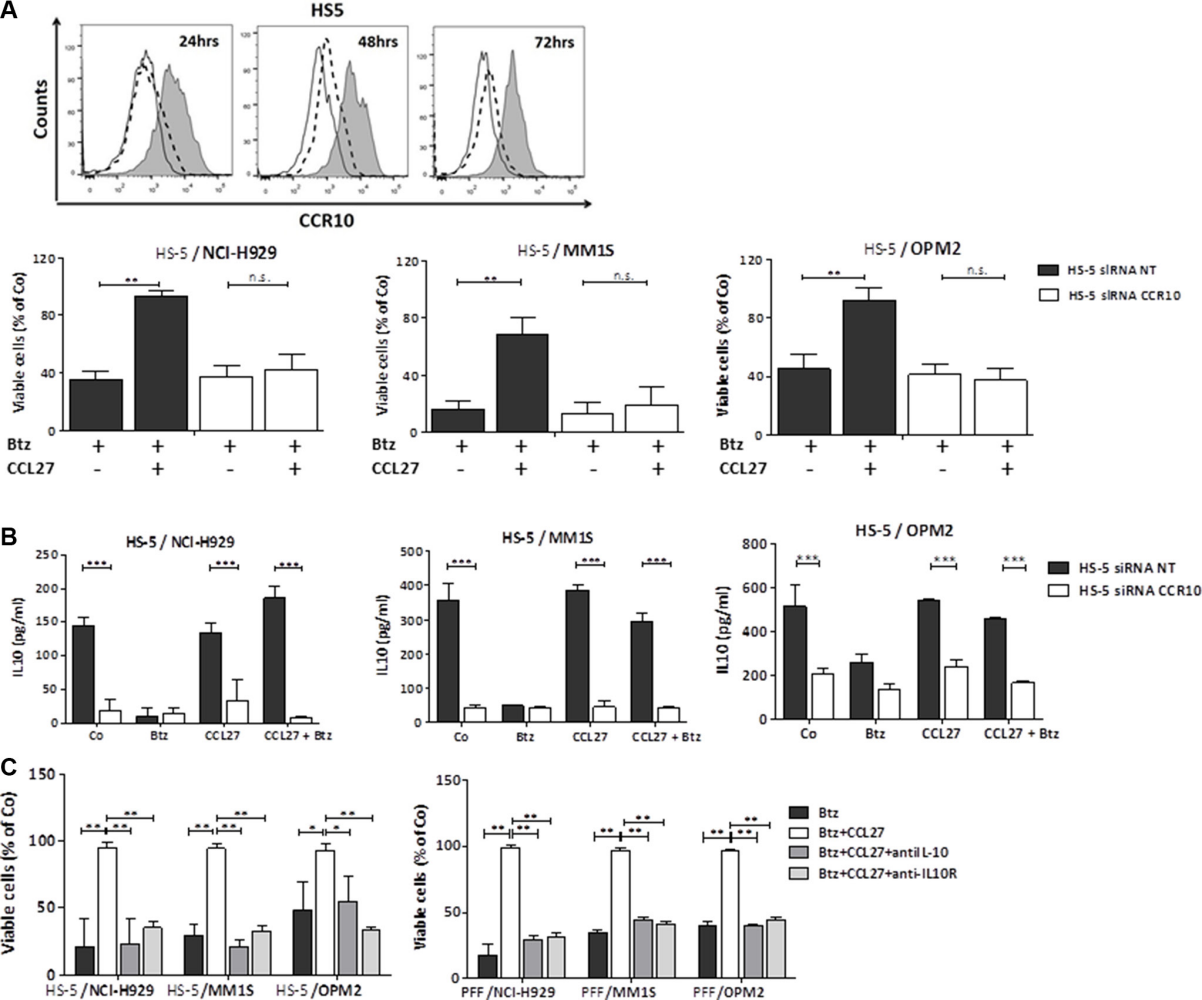
Knocking down stromal CCR10 expression and blocking IL-10 resensitizes myeloma cells to bortezomib (**A**) Time course of CCR10 surface expression in HS-5 stroma cells transfected with 25 nM non-target siRNA control (solid histogram) or transfected with 25 nM CCR10 specific siRNA (open histogram, dashed line) relative to isotype control (open histogram, solid line) was determined by flow cytometry and is shown in the histograms. Graphs: Myeloma cells were cocultured with transfected HS-5 stroma cells (non-targeting siRNA (siRNA NT) and CCR10-specific siRNA (siRNA CCR10), respectively) at a ratio of 2:1, and treated for 48 hrs as indicated (bortezomib 5.2 nM, CCL27 7.9 nM). Viability of myeloma cells is presented as percentage of control (*n* = 4, mean+/−SD; **p < 0.01). (**B**) Myeloma cells were cocultured with transfected HS-5 cells and treated as above. IL-10 levels of the supernatants were measured by Elisa after 24 hrs. Data are shown as the mean ± SD from 4 independent experiments. **p < 0.01; ***p < 0.001; (**C**) Viability of myeloma cells cocultured for 48 hrs with HS-5 cells (left graph) or primary fibroblast (PFF) cells (right graphs) and treated with bortezomib (5.2 nM), CCL27 (7.9 nM), and blocking antibodies against IL-10 and IL-10 receptor (both at 2 μg/well), respectively, is shown as mean of at least 3 independent experiments performed in triplicates, +/− SD. *p < 0.05, **p < 0.01, ***p < 0.001.

Knock-down of CCR10 on HS-5 cells by siRNA resensitized cocultured myeloma cells to bortezomib (Figure 5A). Of note, downregulation of CCR10 per se significantly blocked IL-10 production in cocultures and CCL27 addition did not enhance levels (Figure 5B). Drug resistance was also reversed by the addition of neutralizing antibodies to IL-10 and IL-10 receptor (Figure 5C).

### IL-10 activates survival pathways, counteracts blockade of proteasomal activity and decreases surface immunoglobulin expression of myeloma cells

**Figure 6:**
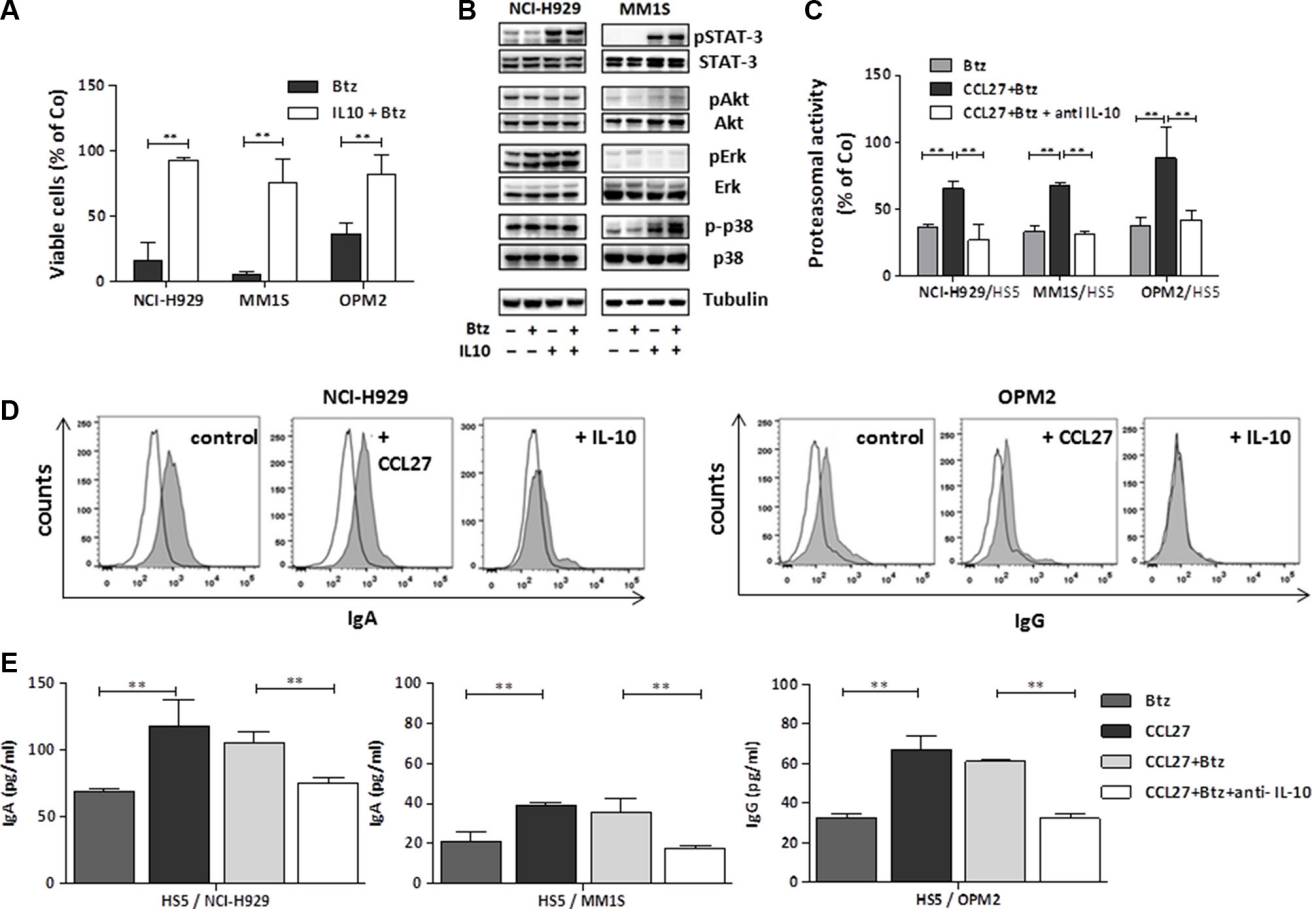
Impact of IL-10 on myeloma cell survival, signalling, proteasomal activity and presentation of surface immunoglobulins (**A**) Myeloma cells (1 × 10^5^ cells/well) were treated with 5.2 nM bortezomib with/without the addition of 5.5 nM IL-10 and cell viability was measured after 48 hrs. Mean percentage of viable cells (of control) +/− SD of 3 independent experiments is shown. **p < 0.01; (**B**) Cell lysates from myeloma cells (1 × 10^6^ cells) treated as indicated for 3 hrs (bortezomib 5.2 nM, IL-10 5.5 nM) were analysed by Western blot for the quoted proteins. Representative blots for NCI-H929 and MM.1S cell line, respectively, are shown. Tubulin is used as loading control. Quantification of band intensities is summarized in Supplemental Table 1. (**C**) Proteasomal activity in cocultured myeloma cells (ratio 2:1) treated for 30 minutes as indicated (bortezomib 5.2 nM, CCL27, 7.9 nM, anti-IL-10 2 μg/well) was measured using Proteasome-Glo^™^ Cell-Based Assays and is shown as percentage activity of untreated cells (*n* = 3, **p < 0.01). (**D**) Surface immunoglobulin expression after 24 hrs incubation with 7.9 nM CCL27 or 5.5 nM IL-10 was measured by FACS. Histograms show one representative analysis out of three per cell line. Open histogram: isotype, solid histogram: specific staining. (**E**) Secreted Immunoglobulin levels (IgA and IgG) in the supernatant of coculture experiments treated as indicated (bortezomib 5.2 nM, anti-IL-10 2 μg/well) for 24 hrs were measured by Elisa and results are shown as mean pg/ml +/− SD (*n* = 3, **p < 0.01).

In further experiments, we found that recombinant IL-10 rescued the malignant plasma cells from bortezomib-induced cell death also when cultured alone, circumventing the need for CCL27 and stromal support (Figure 6A).

IL-10 is known to signal mainly via phosphorylation of STAT-3, which we also confirmed. IL-10 induced p-p38 in MM.1S cells but not in NCI cells and had no significant impact on p-AKT and p-ERK expression in both cell lines. Bortezomib treatment did not alter phosphorylation patterns significantly at the chosen time point (Figure 6B and Supplementary Table 1). The specific rescue from bortezomib-induced cell death by CCL27 led us to further investigate the proteasomal activity of myeloma cells under treatment. Briefly, treatment-related decrease of activity was significantly restored by the addition of CCL27 and this effect was abolished by an IL-10 blocking antibody (Figure 6C). Concomitantly, surface expression of monoclonal immunoglobulin was decreased on IL-10 treated myeloma cells (NCI-H929, IgA myeloma; OPM-2, IgG myeloma) (Figure 6D), whereas we found higher levels of free immunoglobulin in the supernatants of these cells upon CCL27 +/− bortezomib treatment. This effect was blocked by the addition of an anti-IL-10 antibody (Figure 6E).

In summary, we found that CCL27, a novel chemokine in the context of the bone marrow microenvironment, confers myeloma drug resistance to proteasome inhibitors by binding to stromal CCR10 and inducing IL-10, a myeloma survival factor. Enhanced IL-10 levels foster phosphorylation of the survival molecule STAT-3 as well as modulation of proteasomal activity and shedding of immunoglobulins, contributing to myeloma survival under treatment.

## DISCUSSION

Resistance to the proteasome inhibitor bortezomib, which is currently utilized in most therapeutic schemes, is an Achilles heel of myeloma therapy. About one third of myeloma patients are primary refractory to bortezomib treatment while most other patients develop resistance over time [33, 34] with no predictive markers known so far. Here, we discovered that a single chemokine, CCL27, which is upregulated in myeloma patients, induced bortezomib resistance in myeloma cells. The effect was intimately linked to stromal expression of CCR10 and the production of IL-10. Notably, blocking stromal CCR10 expression or IL-10 signalling resensitized the malignant cells to treatment.

From our data, CCL27 emerges as novel chemokine in the context of the bone marrow microenvironment. Its presence in healthy control samples suggests a homeostatic role in this compartment, which has yet to be defined. In this regard, stroma cells might be one source of this chemokine. Enhanced levels in patients correlated with lower overall survival and levels were specifically high in about 40% of primary refractory patients. We hypothesize that the significant rise in CCL27 expression found in these patients facilitates oligomerization at the receptor level leading to specific, pro-tumor signaling. Oligomerization is often required *in vivo* for functional output of chemokine signaling [35], which might include cytokine induction. CCL27 has been described to oligomerize over a narrow concentration range, which is known to be relatively high [36]. In line with this, we found that CCL27 levels in the range of healthy donors (up to 3000 pg/ml) could not protect myeloma cells from bortezomib-induced cell death *in vitro*. However, levels of the defined “high expressors” (same or higher as 5000 pg/ml) led to drug-resistance in our models. Therefore, in the patient setting, the selection of high producer myeloma cell clones and/or the observed upregulation of CCL27 in myeloma cells upon bortezomib-treatment could tip the balance in favour of the tumor. Importantly, many other cell types are involved in the microenvironmental crosstalk in myeloma (reviewed in [37]) with some of them (e.g. specific subtypes of T cells and dendritic cells) also being responsive to CCL27. Other cell types might also modulate levels of soluble CCL27 and these putative interactions could essentially contribute to the observed effect *in vivo*. Further in depth-investigations are certainly needed.

In any case, stromal CCR10 expression is a prerequisite for the observed rescue. Therefore, targeting this receptor could be a novel and efficient therapy for resistant and primary refractory patients. First data on the CCR10 antagonist POL7085, a protein epitope mimetic, which was recently successfully used in a model of allergic eosinophilic airway inflammation [38] might pave the way for an application in multiple myeloma. Side effects should be manageable since CCR10 knock out mice displayed mild defects of plasma cell [19] and skin-specific T cell migration [39] and showed no obvious phenotype.

Concerning the involved pathways, CCL27 is known to be activated by the NF-κB pathway [40], and should thus be down-regulated by bortezomib treatment. In our experiments, we observed upregulation of CCL27 and a CCL27-induced stabilization of IL-10 in response to bortezomib, which is totally unexpected and in contrast to current data [32, 41]. Moreover, we could show that CCL27 itself induces NF-κB signaling in stroma cells and this signaling might contribute essentially to high IL-10 levels. Since NF-κB constitutes also a major survival pathway of myeloma cells and their microenvironment in general, this counterregulation by CCL27 warrants further detailed investigations.

In our experiments, IL-10 plays an essential role in the transduction of survival. This cytokine has been shown to induce myeloma cell survival and growth and to confer sensitivity to various other gp130-activating cytokines, including IL-6 [30, 42]. Targeting IL-10 could be of additional interest in the context of tumor immune evasion, since it is known as an immunosuppressive cytokine [43]. However, *in vivo*, IL-10 levels are not detectable in the majority of patients with established myeloma disease and expression appears to be mostly restricted to patients suffering from plasma cell leukemia [42, 44]. Recent data utilizing an ultrasensitive Elisa approach, however, support an *in vivo* impact of IL-10 also in myeloma, although concentrations measured were in the low picogram range [45]. It is likely that microenvironmental IL-10 is immediately utilized by the myeloma cells and therefore efficient targeting might be hard to accomplish. In contrast, targeting chemokine/receptor crosstalk is feasible and therapeutically relevant, as has been shown e.g. for CXCL12/CXCR4 also in myeloma [46, 47].

In summary, we suggest that targeting the CCR10/CCL27 crosstalk in the myeloma microenvironment could contribute to the development of tailored therapeutic interventions bringing multiple myeloma to a chronic, manageable disease or even to cure.

## MATERIALS AND METHODS

### Patient samples

Plasma from bone marrow aspirates of the proximal femur during hip arthroplasty were collected through routine analysis of healthy donors (*n* = 16) at the Department of Orthopedic Surgery, University Hospital Innsbruck. These healthy subjects were screened for the presence of viral and bacterial infections or any bone abnormalities and did not receive immunomodulatory drugs or suffer from diseases known to influence the immune system, including autoimmune diseases or cancer. Bone marrow aspirates from the posterior iliac crest of myeloma patients (*n* = 45) were taken at the Department of Internal Medicine, Innsbruck Medical University. In addition, untreated samples from patients that were found to be refractory to bortezomib at first line treatment (*n* = 12) and at later lines (2–11; *n* = 18), respectively, were collected at the Department of Internal Medicine, University Hospital Brno, Czech Republic, and analyzed retrospectively. For the standardized sample preparation, heparinized bone marrow (5 ml, 10 U sodium-heparin/ml of sample) was centrifuged within 1 hour at the Tyrolean Cancer Research Insitute in Innsbruck and at Brno Hospital, respectively, at 400 g for 10 minutes and plasma was immediately stored at −80°C until further analyses. The study was conducted in accordance with the Declaration of Helsinki and ethical approval was given by the local Ethics Committee (approval numbers UN4402, UN3263, UN5064 (Innsbruck) and 16/2011 (Brno). Patients as well as healthy donors provided written informed consent. Primary myeloma cells were purified using CD138 microbeads (Miltenyi Biotec, Germany) following manufacturer's protocol and primary stroma cells were collected from bone marrow aspirates due to their adherence on plastic and flow cytometric analysis of their phenotype (CD90+/CD45−).

### Cell lines, cell culture, and reagents

Human myeloma cell lines NCI-H929 and OPM-2 were purchased from DSMZ (Braunschweig, Germany), the human stroma cell line HS-5 from American Type Culture Collection (Rockville, USA), and primary foreskin fibroblasts from Promocell company (Germany). MM.1S were a generous gift from Dr. N. L. Krett [48] (Northwestern University, Chicago, USA). Cell lines were routinely grown in RPMI-1640 (Lonza, Switzerland) with 10% fetal calf serum (Sigma, USA), 100 μg/ml L-glutamine and 100 U/ml penicillin-streptomycin (Life Technologies, UK) at 37°C with 5% CO_2_. For MM.1S, fungizone (Fisher Scientific, Austria) was added as described [48]. Cells were obtained from DSMZ and ATCC in 2012 and expanded within 6 weeks. Frozen aliquotes were further utilized for no more than 3 months. Additionally, cell lines were routinely fingerprinted at the end of the experiments to re-confirm their origin (STR-method, Institute of Legal Medicine, Medical University of Innsbruck) and tested for mycoplasma negativity.

Other purchased reagents include Bortezomib, Carfilzomib (Sellekchem, USA), MG132 (Sigma, USA), recombinant human CCL27, CCL28 (R&D Systems, USA), IL-10 (eBioscience, USA) and the anti-human antibodies for IL-10 (clone #JES3-9D7, eBioscience, USA), IL-10 receptor (clone #3F9, Biolegend, USA), CD90 (Acris, USA), CD45, CD38, IgA, and IgG (Miltenyi Biotec, Germany), as well as CCR10 (clone #314305; R&D Systems, USA).

### Protein arrays

Customized chemokine arrays (Ray Biotech, USA) were used to semiquantitatively analyze myeloma cell line supernatants and bone marrow plasma samples. Customized arrays containing antibodies against 107 chemokines, cytokines and growth factors were utilized for the analysis of coculture supernatants following exactly the manufacturer's protocol. Arrays were scanned with GenePixx 4000B microarray scanner (Molecular Devices, USA) and signal intensities at 532 nm were evaluated.

### Elisa

Sandwich Elisa to measure CCL27, CCL28, IL-10, (R&D systems, Germany), immunoglobulins A and G (Cell Biolab, Inc., USA) and green fluorescent protein (GFP; eBioscience, USA), respectively, were performed following the manufacturers' protocols. The optical density was measured using benchmark microplate reader (Bio-rad laboratories, USA).

The amount of DNA-bound and thus activated NF- kB in nuclear extracts (prepared utilizing Nuclear Cell Extraction Kit, Active Motif) of HS-5 stroma cell line and myeloma cell lines NCI-H929, OPM-2, and MM.1S was quantified by Elisa using the TransAM NF-kB p65 Transcription Factor Assay kit (Active Motif North America, Carlsbad, CA) according to manufacturers' protocol and as described before [26].

### Flow cytometry

Surface expression of CCR10, IgA and IgG (Miltenyi Biotec, Germany) was measured by flow cytometry. Flow cytometry was performed on a FACS Canto II flow cytometer with subsequent data analysis using FACS Diva software 7.0 (BD Biosciences, USA).

### Cell adhesion assay

The adhesion of myeloma cells was performed using cellix flow system under shear stress. Vena8 biochips (Cellix Ltd, Dublin, Ireland) were coated with fibronectin+/−CCL27 and +/−CCL28, respectively, blocked with 0.2% BSA for 30 mins at room temperature and washed once with Hank's BSS medium. The cells were loaded into the coated channels at shear stress of 0.5 dynes/cm^2^ using Mirus nanopump (Cellix) and allowed to sit on the layer for 10 mins. Then the flow was started and continued with different shear stress of 0.5 dynes/cm^2^, 0.1 dynes/cm^2^ and 0.2 dynes/cm^2^ for every 30 secs. At different time points, images were recorded and analyzed by DucoCell software (Cellix).

### Transwell migration assay

Myeloma cell migration was performed using an 8 μm pore sized transwell system (Corning costar, USA) coated with fibronectin (100 ng/ml). Myeloma cells at a concentration of 1 × 10^5^ were placed on the upper chamber and the lower chamber was filled with media with or without chemokines (7.9 nM and 8.1 nM). After 8 hrs, the medium in the lower chamber along with the cells were collected and counted by FACS for 1 min. Migration rate was calculated as % increase over control.

### Proliferation assay

Proliferation was measured by ^3^H-thymidine incorporation assays. Myeloma cells (1 × 10^4^ cells/well) were seeded into a 96-well plate with/without the addition of chemokines as described and 1 μCi of ^3^H-thymidine was added to each well for the last 16 hrs of a 72 hrs incubation period. Cells were harvested onto glass fibre filters, scintillation liquid was added and radioactivity was measured using a Scintillation counter (Beckman Coulter, model LS-6500). Experiments were repeated at least 3 times in quadruplicates and statistical analyses were performed utilizing paired Student's *T*-test.

### Apoptosis assay

Myeloma cells (1 × 10^5^/well) were seeded in triplicates in 96-wells and incubated as indicated. In coculture experiments, myeloma cells were seeded onto 50.000 HS-5 stroma cells, i.e. in a ratio of 2:1. Survival of myeloma cells was evaluated by flow cytometry utilizing Annexin-V-APC and 7-AAD staining (BD Biosciences, USA) of gated myeloma cells (CD38high/CD45 low-neg).

### Ex ovo chicken chorioallantoic membrane assay (CAM assay)

The CAM assay used in this study was essentially performed as described previously [26]. We performed two settings: first, collagen onplants containing eGFP-transfected myeloma cells and primary fibroblasts were cultured with/without the addition of CCL27, bortezomib and combinations thereof, respectively. From these xenografts, protein was prepared and tumor load was determined by anti-GFP-Elisa. Second, we seeded onplants as above utilizing myeloma cells without eGFP-transfection. For histological analysis, onplants were excised, fixed in 4% paraformaldehyde solution and further processed for paraffin sectioning and staining. Tumor growth was analyzed by staining proliferating cells with anti-human Ki-67 antibody. Tumor area was determined using the ImageJ program (NIH, USA).

### Proteasome activity assay

Proteasomal activity was measured according to manufacturer's protocol using Proteasome-Glo^TM^ Chymotrypsin-Like Cell-Based Assays (Promega, USA). Luminescence was detected using a luminometer (PerkinElmer, USA) and % activity was calculated according to untreated control.

### CCR10 silencing in stromal cells

HS-5 stroma cells were transfected with 25nM of a pool of 4 different siRNAs specific for CCR10 or non-target control siRNA (Dharmacon, USA) using turbofect transfection reagent (Thermo Fisher Scientific, USA) according to the manufacturer's protocol. After 24, 48 and 72 hrs, respectively, cell viability and CCR10 expression was measured by flow cytometry. Cells were seeded after 24 hrs in 96-well plates and utilized for coculture experiments.

### Western Blot

One million myeloma cells were treated with/without bortezomib (5.2 nM) and IL-10 (5.5 nM) for 3 hrs. Cell lysates were examined by western blot analysis using anti-human antibodies for p-STAT-3, p-Akt, Akt, p-p38, p38, p-Erk, Erk (Cell signaling, USA), STAT-3 (Santa Cruz) and alpha-tubulin (Merck Millipore, Germany).

### Statistical analyses

For all datasets displaying Gaussian distribution an unpaired *t*-test was used for analysis, otherwise MannWhitney *U* test was applied. *P* values < 0.05 were considered as statistically significant (*p < 0.05; **p < 0.01 and ***p < 0.001; n.s. not significant). ROC analysis was used to define cut off for high and low expression of CCL27, respectively. All analyses were performed utilizing GraphPad Prism 5 software (GraphPad Software, Inc, USA). Kaplan-Meier curves were applied for survival analysis. SPSS statistics was used for multivariate analysis.

## SUPPLEMENTARY MATERIALS


