# Supplementary material for: CCR10/CCL27 crosstalk contributes to failure of proteasome-inhibitors in multiple myeloma
Source: Oncotarget. 2016 Oct 8;7(48):78605–18. doi: 10.18632/oncotarget.12522 (PMC5346663; doi:10.18632/oncotarget.12522)
Supplement: Supplementary file 1 [file oncotarget-07-78605-s001.pdf]

CCR10/CCL27 crosstalk contributes to failure of proteasome-inhibitors in multiple myeloma

Supplementary Materials

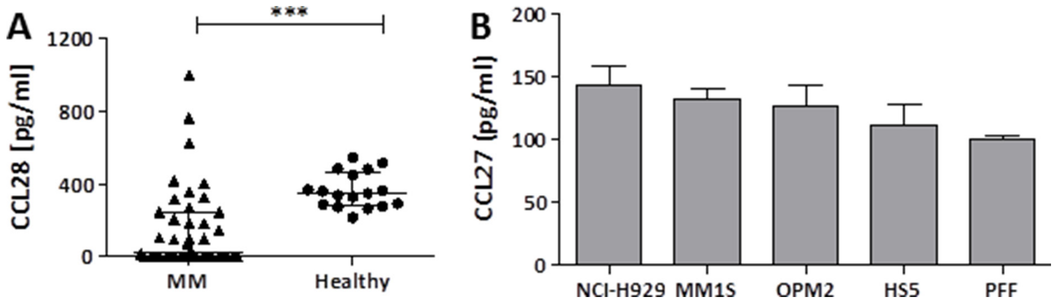

Supplementary Figure S1: Chemokine Elisa of CCR10 ligands. (**A**) CCL28 levels were measured by Elisa in bone marrow plasma of healthy donors and myeloma patients and results are shown in pg/ml (median +/− IQR). ***$p < 0.001$; (**B**) CCL27 levels were assessed by Elisa in cell culture supernatants of myeloma cells and stroma cells, respectively. Cells (1×10^6) were cultured for 24 hrs in complete RPMI-1680 media with 1% FCS and values are depicted in mean pg/ml +/− SD.

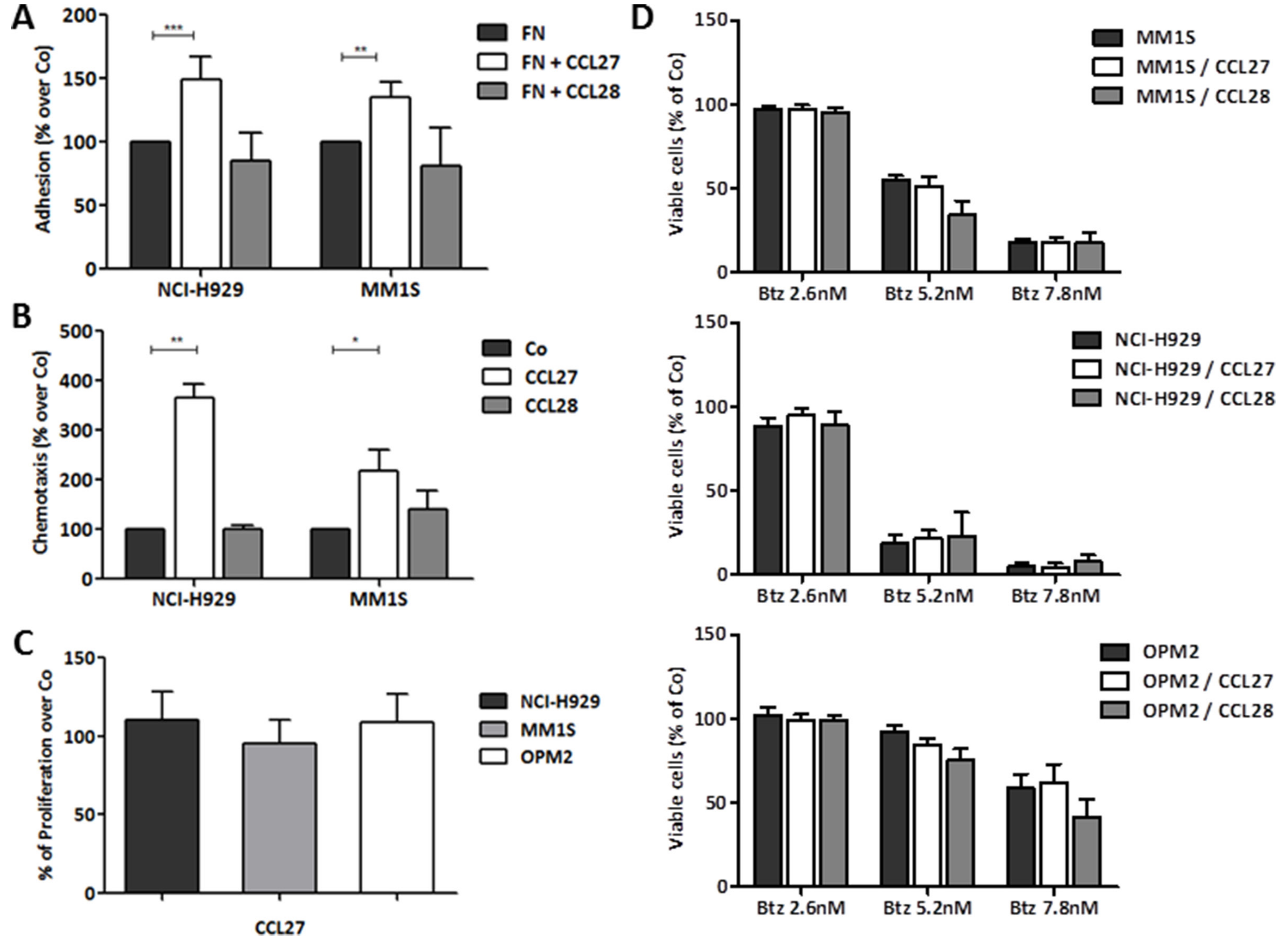

Supplementary Figure S2: Adhesion, migration, proliferation and bortezomib-induced cell death of myeloma cell lines in response to CCL27 and CCL28. (**A**) Adhesion of myeloma cells was measured utilizing a flow system and percentage of adherent cells was calculated as percentage of control (no chemokine). FN: fibronectin; CCL27 7.9 nM, CCL28 8.1 nM; $**p < 0.01$, $***p < 0.001$; (**B**) Migration in response to chemokines was analyzed via a transwell-system using 100.000 cells per assay, transwells with 8 µm pore size, 8 hrs transmigration time, and CCL27 (7.9 nM)/ CCL28 (8.1 nM). Migrated cells were counted by flow cytometry (events/1 min) and calculated as percentage of control (no chemokine). (**C**) ^{3}H-thymidine incorporation assay was performed to assess putative changes in proliferation rates of myeloma cells in response to chemokine CCL27 (7.9 nM, total 72 hrs incubation). All assays were performed at least in triplicates at 3 different time points. (**D**) Cell death of myeloma cells (1×10^{5}/well, as in coculture experiments) in response to 48 hrs-treatment with different concentrations of bortezomib with/without CCL27/CCL28 addition (7.9 nM and 8.1 nM, respectively) was analysed by flow cytometer by staining cells with AnnV/7-AAD. Percentage of cells alive (AnnV/7-AAD negative) with respect to untreated cells are displayed. For all assays, students *T*-test was performed; $*p < 0.05$, $**p < 0.01$, $***p < 0.001$.

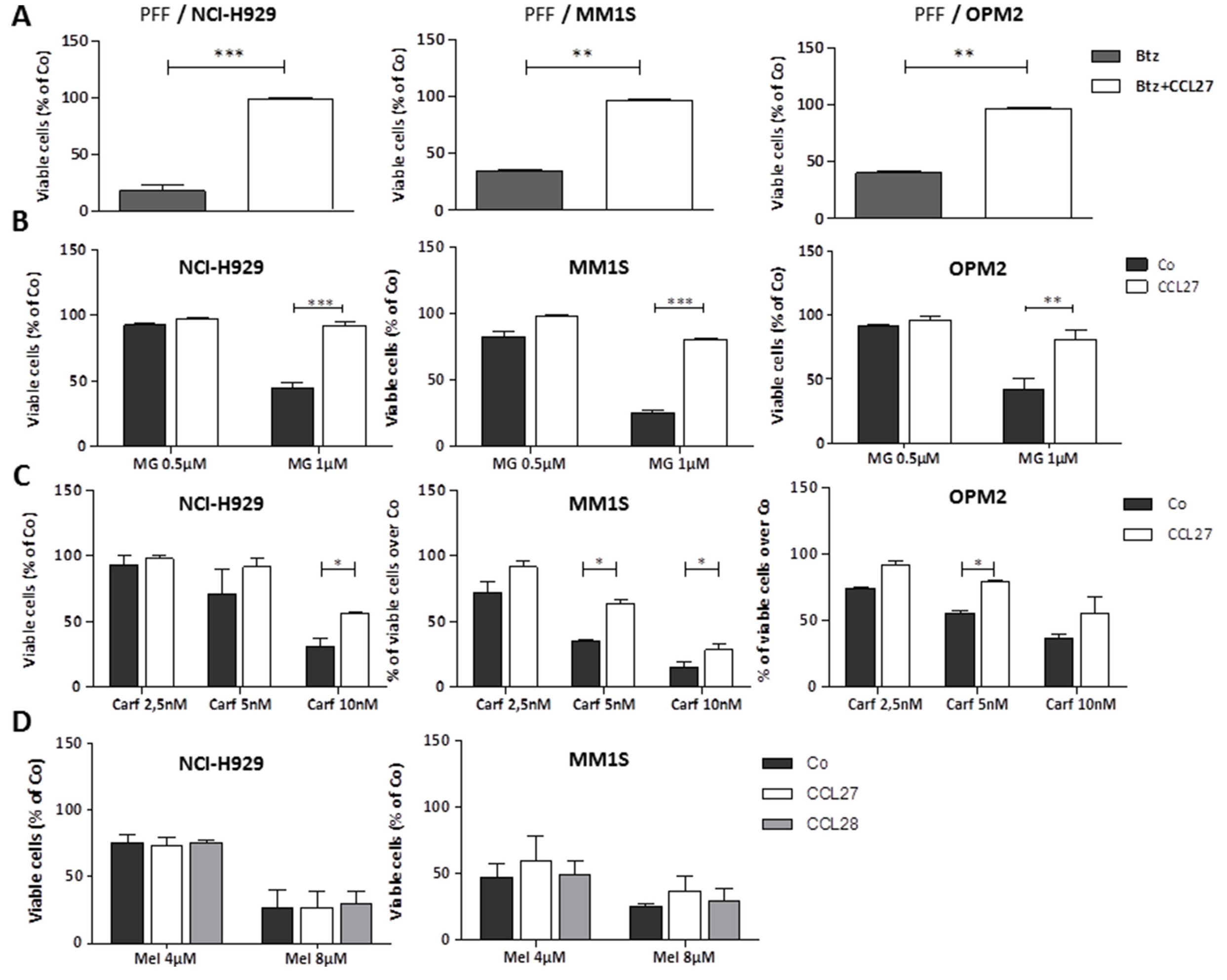

Supplementary Figure S3: Coculture and CCL27 rescue myeloma cells from cell death induced by proteasome inhibitors. (**A**) Myeloma cells were treated in coculture with primary fibroblasts (PFF) as indicated (ratio 2:1, bortezomib 5.2 nM, CCL27 7.9 nM). Viable cells were defined as AnnV/7-AAD negative cells using a flow cytometer. Similar, viability of cocultured myeloma cells was measured under treatment with (**B**) MG-132 (MG) as another reversible proteasome inhibitor, (**C**) carfilzomib (Carf) as an irreversible proteasome inhibitor and (**D**) melphalan (Mel) as an alkylating agent at indicated concentrations. Viability of cells was measured as above, **p* < 0.5, ***p* < 0.01, ****p* < 0.001.

Supplementary Table S1: Quantification of western blots

NCI					MM1.S				
	Co	Btz	IL-10	Btz+IL-10		Co	Btz	IL-10	Btz+IL-10
p-STAT-3	1.0	0.8	1.8	1.6	p-STAT-3	1.0	2.0	190.5	195.3
STAT-3	1.0	1.0	1.0	1.0	STAT-3	1.0	1.4	1.3	1.1
p-Akt	1.0	0.8	0.9	0.8	p-Akt	1.0	1.8	2.1	2.2
Akt	1.0	0.9	0.9	1.0	Akt	1.0	1.6	1.7	1.5
p-Erk	1.0	1.2	1.3	1.4	p-Erk	1.0	1.8	0.8	1.1
Erk	1.0	1.0	1.1	1.3	Erk	1.0	0.9	0.8	0.8
p-p38	1.0	1.1	0.9	1.1	p-p38	1.0	1.2	1.7	2.9
p38	1.0	1.1	1.3	1.3	p38	1.0	1.3	1.6	1.3

Myeloma cell lines NCI-H929 and MM.1S were treated with bortezomib, IL-10, and both, respectively. Intensities of bands were normalized to the expression of housekeeping gene tubulin. Thereafter, fold changes in intensities in treated samples compared to untreated control was calculated and is summarized in the table.
